# Primary mental health care in India for older people: stigmatization and community-orientation

**DOI:** 10.1093/eurpub/ckac129.719

**Published:** 2022-10-25

**Authors:** T Kafczyk, K Hämel

**Affiliations:** Health Services Research and Nursing Science, School of Public Health, Bielefeld University, Bielefeld, Germany

## Abstract

**Background:**

In India, older persons are among the fastest growing population groups that are particularly vulnerable to an impaired mental health. Strengthening public primary mental health care (PMHC) has been one of the political priorities in recent years, including efforts to provide more old age-inclusive care. Many factors play a role in shaping equitable access to PMHC. In this study, we focus on how social norms and perceptions of mental health in old age on the community-level facilitate or hamper access to inclusive PMHC.

**Methods:**

Semi-structured interviews with key informants (n = 14) from the fields of mental health and old age policy and practice have been conducted and analyzed.

**Results:**

The interviewees describe barriers and opportunities towards old-age inclusive and community-oriented PMHC that go beyond the availability of services. The stigmatization of mental health - especially in old age - is a major barrier that is deeply rooted within the family and community care system. While experts stress the relevance of relatives in fostering mental health of older people, a lack of awareness of facets of mental health and a fear of stigmatization within communities prevents older persons and their family caregivers to seek formal mental healthcare. Moreover, experts describe an increasing disintegration of family support systems in rural and urban areas. Therefore, a more prominent role for outreach-support provided by community health workers (CHWs) is suggested. However, according to experts, transforming the scope of practice of CHWs to include mental healthcare for older persons is still predicted to be a major challenge.

**Conclusions:**

To improve equitable access to and participation of older persons and their families in PMHC, the perceptions and attitudes towards mental health and old age need to be considered.

